# Naming internal insertion alleles created using CRISPR in *Saccharomyces cerevisiae*

**DOI:** 10.17912/micropub.biology.001258

**Published:** 2024-08-08

**Authors:** Xheni Mucelli, Linda S Huang

**Affiliations:** 1 Biology, University of Massachusetts Boston

## Abstract

The budding yeast *Saccharomyces cerevisiae* is a powerful model organism, partly because of the ease of genome alterations due to the combination of a fast generation time and many molecular genetic tools. Recent advances in CRISPR-based systems allow for the easier creation of alleles with internally inserted sequences within the coding regions of genes, such as the internal insertion of sequences that code for epitopes or fluorescent proteins. Here we briefly summarize some exisiting nomenclature standards and suggest nomenclature guidelines for internal insertion alleles which are informative, consistent, and computable.

**Figure 1. BIM1-S334_N335insENVY contains an internally inserted ENVY sequence between the codons for amino acids S334 and N335:**
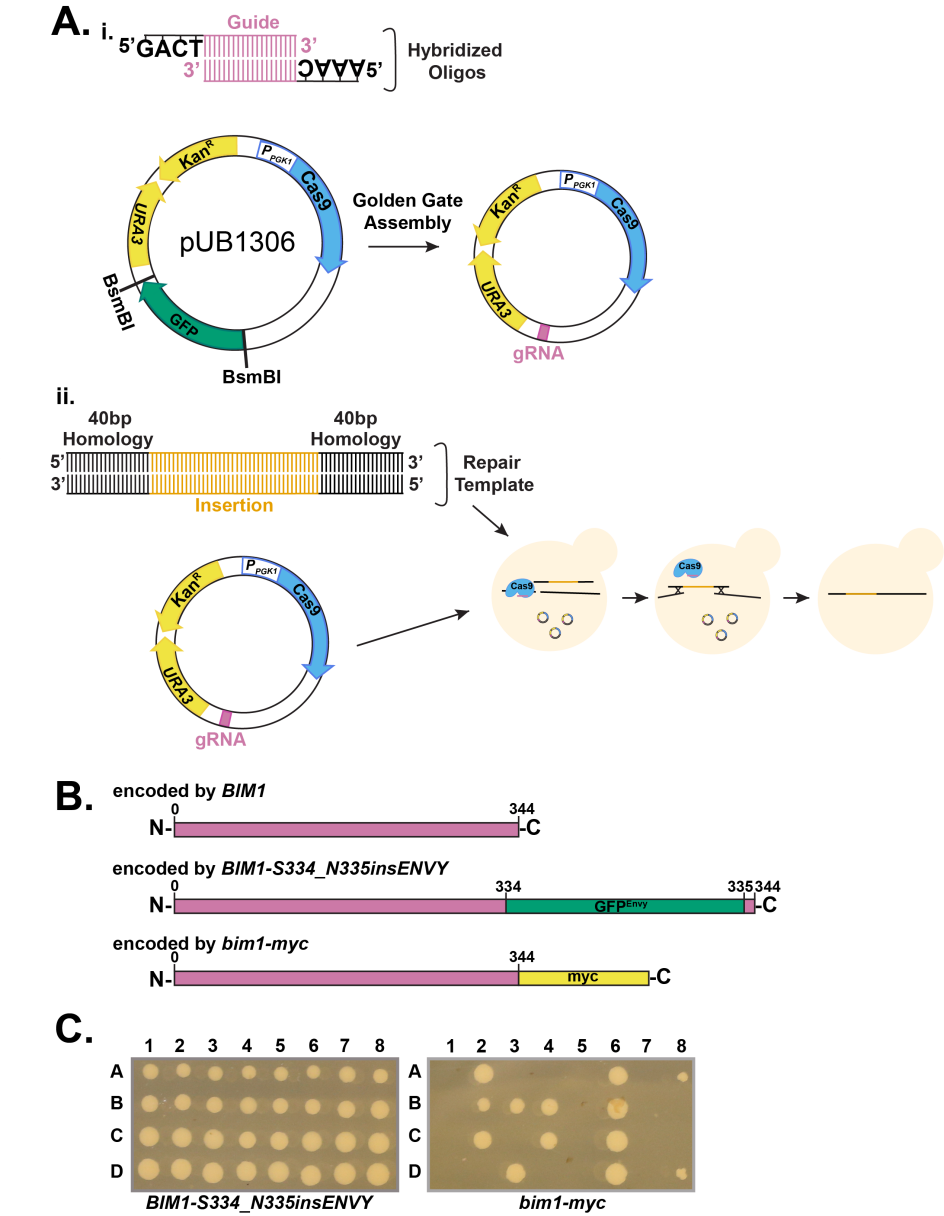
(A) Methods for creating an insertion using CRISPR-Cas9 in budding yeast. *i* ) Guide RNA (gRNA) sequences are inserted into pUB1306, replacing the GFP between the BsmBI sites. Key elements drawn: Cas9 (blue) under control of the *PGK1* promoter ( *P _PGK1_* ), genes for selection ( *URA3* and Kan ^R^ ; yellow), and gRNA (pink). *ii* ) CRISPR process to insert DNA sequence within the genome. The repair template along with the plasmid encoding the guide sequence and Cas9 are co-transformed into yeast. After the Cas9 cut in the genomic DNA, the yeast will repair the double strand break, leading to insertion of new sequence within the gene. Counterselection against the *URA3* marker using 5-FOA leads to loss of the plasmid encoding Cas9 and the guide. (B) Depiction of the proteins encoded by the genes *. BIM1* and *BIM1-S334_N335insENVY* encode functional gene products while *bim1-myc* does not complement *.* (C) *BIM1-S334_N335insENVY* complementation. Eight tetrads dissected from *BIM1-S334_N335insENVY* (LH1158) and *bim1-myc* (LH1161). Each numbered column represents the four spores from the same parent (and thus, each column represents a different tetrad that has been dissected) while each row (labeled A, B, C, and D) indicates where the spores were placed to examine germination and the ability to form a colony.

## Description

**Introduction**

The budding yeast *Saccharomyces cerevisiae* is a prominent model for investigating basic eukaryotic cell biology. One beneficial characteristic of studying *S.* *cerevisiae* is the powerful molecular genetic tools available to modify the genome (Botstein and Fink 2011) . The propensity of *S. cerevisiae* to undergo homologous recombination has allowed for easy insertions of sequences into the genome (Orr-Weaer et al *.* 1981; Orr-Weaver et al *.* 1983; Vanderwaeren et al *.* 2022). There are many tools for PCR-mediated insertion of sequences at the 5’ and 3’ ends of genes (i.e., Schneider et al. 1995; Wach et al *.* 1997; Bahler et al *.* 1998; Longtine et al. 1998; Knop et al. 1999; Janke et al *.* 2004; Sheff and Thorn 2004; Lee et al. 2013) which allow for the manipulation of endogenous loci. The insertions of promoter sequences, epitope sequences, or fluorescent protein sequences at the 5’ and 3’ ends of genes are readily achievable using minimal (35-50 base pairs) homology, which are added by PCR to the sequence to be inserted.

Insertions of epitopes or fluorescent proteins at the N- or C-termini of proteins can sometimes disrupt protein function. In these cases, sometimes the insertion of the epitope or fluorescent protein internally within the protein can create a functional protein (for example, Woods et al *.* 2015; Wu et al *.* 2015; Lang et al *.* 2015; Anand et al *.* 2017; Kornakov et al. 2023). In the past, insertion alleles have been notated using different methods, some which did not clearly indicate the position of the insertion. The creation of internal insertion alleles can now be more easily done due to the adaptation of CRISPR technologies in *S. cerevisiae* (DiCarlo et al *.* 2013; Laughery et al *.* 2015; Anand et al *.* 2017; Levi et al *.* 2020), and multiple insertions at different sites for a single gene can also be made. Some of these insertion alleles may be functional while others may not. Thus, there is a need to update existing nomenclature guidelines for internal epitope/fluorescent protein insertion alleles that are informative while maintaining consistency, computability, and clarity.

The *Saccharomyces* Genome Databases (SGD) has updated nomenclature guidance with advancements in knowledge and techniques (Cherry et al. 1998; Engel et al *.* 2022; Wong et al *.* 2023). Recently, other model systems have also reviewed and updated nomenclature standards within their field to increase recognition of alleles for data mining and machine learning (Lera-Ramirez et al *.* 2023). Here, we briefly review some of the relevant existing nomenclature guidelines in *S. cerevisiae* and suggest a methodology for naming alleles that insert epitopes/fluorescent protein coding sequences within gene coding regions.

**Results and Discussion**

*Existing gene nomenclature in S. cerevisiae*

The nomenclature guidelines for *S. cerevisiae* were summarized shortly before the publication of the yeast genome sequence in 1996 (Cherry 1995) , and the majority of these rules are still utilized today. Gene names are italicized and comprise three letters followed by a number (i.e., *XYZ1* ). These symbols are all uppercase if the allele is dominant and lowercase if the allele is recessive; wild type alleles are typically uppercase. When genomic alterations are made, alleles are annotated by the addition of a symbol to indicate the nature of the alteration: disruptions are annotated with a double colon (::), deletions can be indicated with a lower case delta (∆), epitopes or fluorescent proteins which are inserted at the endogenous locus are denoted with a dash (-) and the addition of auxotrophic or antibiotic resistance markers linked to the allele are indicated with a single colon (:). Examples of all of these are provided in Table 1 using the hypothetical *XYZ1* gene as an example.

**Table 1. Nomenclature examples (based on Cherry 1995)**

**Table:** 

**Gene**	**Description of use**
*XYZ1*	Wild type or dominant genes
*xyz1*	Mutant/recessive genes that may produce protein
*xyz1∆*	Complete open reading frame deletion
*xyz1::URA3*	Disruption, replacing open reading frame with *S. cerevisiae URA3*
* xyz1::URA3 ^K.l.^*	Disruption, replacing open reading frame with with *K. lactis URA3*
*XYZ1-GFP*	Complementing extreme C-terminal addition of GFP
*XYZ1-GFP:TRP1*	Complementing extreme C-terminal addition of GFP that includes a 3’ selectable marker ( *TRP1* )
*xyz1-GFP*	Noncomplementing extreme C-terminal addition of GFP
*GFP-XYZ1*	Complementing extreme N-terminal addition of GFP
*GFP-xyz1*	Noncomplementing extreme N- terminal addition of GFP

*Naming insertion alleles with wild type functions*

There are currently no guidelines for naming *S. cerevisiae* alleles created by an internal epitope or fluorescent protein insertion, resulting in alleles being named using multiple methods. For example, insertion alleles have been denoted using a superscript SW (representing sandwich) after the fluorescent protein (Bendezu et al. 2015; Wu et al. 2015; Woods et al. 2015), by using a caret (^) between the gene name and the epitope (Lang et al. 2015) , or by using a superscript WT (representing wild type, as for that particular insertion allele did complement while a C-terminal insertion did not) (Kornakov et al. 2023) .

Although addition of the Green Fluorescent Protein ( *GFP* ) gene at the 3’ end of *BIM1* created an allele that did not complement, insertion of *GFP* in between the codons encoding Serine (S) 334 and Asparagine (N) 335 created a functional *BIM1* allele. This insertion allele can support cell viability in a Spindle Assembly Checkpoint deficient background and complement *BIM1* function, as assessed by temperature, hydroxyurea, and benomyl hypersensitivities (Kornakov et al. 2023) .

We recreated this *BIM1* insertion allele in the SK1 (Kane and Roth 1974) strain background using a CRISPR-based strategy (Anand et al. 2017; Sawyer et. al 2018; Schlissel and Rine 2019; Figure 1A ), modifying the genome to insert sequences encoding for the GFPvariant, Envy (Slubowski et al. 2015) in between the codons for S334 and N335 of Bim1 ( Figure 1B ). This insertion alelle in the SK1 background sporulates like wild type and leads to 100% spore germination, unlike the *bim1-myc* allele (Seitz et al. 2023) , which results in a variable spore germination and cell growth phenotypes ( Figure 1C ).

We propose to name this insertion allele *BIM1-S334_N335insENVY* . We chose this name to maintain computability, while providing valuable information regarding the allele. The hyphen between the gene name *(BIM1* ) and the portion denoting insertion site indicates that we have appended a fluorescent protein. All capital letters are used in the gene name, as this particular insertion allele complements function and behaves like the wild type allele. The site of insertion is denoted using both the amino acid number and the single letter code for the amino acid; using the amino acid code indicates that the number refers to protein and not genome coordinates. Specifying the amino acid name helps to maintain stable and unambiguous identification of the insertion site. To denote this an insertion allele, we use “ins”; carets and superscripts, which were previously used, can be problematic in databases.

This nomenclature scheme considers the naming conventions set up by the HGVS Nomenclature for naming human genome variants (den Dunnen et al. 2016; see https://hgvs-nomenclature.org/ ) with some minor modifications. Instead of using genome coordinates, we use amino acid coordinates. First, amino acid coordinates are more familiar to most yeast biologists who are studying the functions of genes and gene products. Second, because there are several commonly used strain backgrounds used to study *S. cerevisiae* , these strain backgrounds would have different genomic coordinates but share more similarity at the amino acid level. Finally, we chose to use the single letter amino acid code instead of the three letter code for brevity.

*Other related scenarios for internal epitope insertions*

Sometimes, insertion of an epitope or fluorescent protein can lead to loss of gene function. If this were the case for gene *XYZ1* that is internally modified between amino acid residues proline (P) 100 and tyrosine (Y) 101, we suggest to name this type of allele *xyz1-P100_Y101insGFP* *.* In this case, the lowercase gene name denoting this is a recessive allele of gene *XYZ1* that has GFP coding sequences inserted between amino acids P100 and Y101.

Sometimes an epitope may replace a region of the gene, instead of a simple insertion. For example, if GFP replaced a protein domain found between amino acids Leucine (L) 350 and Phenylalanine (F) 450 in protein Xyz1, the allele could be called *xyz1-L350_F450insGFP* if this allele had a recessive phenotype and *XYZ1-L350_F450insGFP* if this allele were fully functional. These addition to the existing nomenclature guidelines are in Table 2.

Table 2. Nomenclature for insertion alleles

**Table:** 

**Gene**	**Description of use**
*BIM1*	Wild type or dominant genes
*BIM1-S334_N335insENVY*	Complementing internal insertion of sequences encoding the GFP variant Envy
*XYZ1*	Wild type hypothetical gene
*XYZ1-P100_Y101insGFP*	Complementing internal insertion of sequences encoding GFP
*xyz1-P100_Y101insGFP*	Noncomplementing internal insertion of sequences encoding GFP
*XYZ1-L350_F450insGFP*	Complementing allele that replaces part of a gene with sequences encoding GFP
*xyz1-L350_F450insGFP*	Noncomplementing allele that replaces part of a gene with sequences encoding GFP

The advancement of technologies for genetic manipulations can require an update to nomenclature guidelines. Ideally, the use of proper nomenclature will provide important information both to the reader and for the purposes of text mining and computability. The nomenclature guidelines we suggest allows for the ability to search for insertion alleles (due to the use of “ins”) and also provides an understanding of where the insertion is and whether it is a complementing allele or one that is loss of function/hypomorphic.

## Methods

**Materials and Methods**

All strains used in this study are derivatives of the SK1 background often used for studying the sporulation process (Kane and Roth 1974) . Standard genetic methods were used to create and propagate strains (Rose and Fink 1990) . Complete genotypes, plasmids, and primers can be found in Tables 3-5.

Construction of the *bim1-myc* allele was previously described (Seitz et al. 2023) . The non-complementing *bim1-myc* homozygous strain (LH1161) was created by taking *MAT* ***a*** *bim1-myc* , backcrossing it to wild type *MATα* (LH176) and dissected to obtain a *MAT* ***a*** *bim1-myc* and *MATα bim1-myc* strain (LH1159 and LH1160), and mating these two strains.

To create pXM27, guide RNA (gRNA) sequences were inserted into pUB1306 to replace the GFP cassette. OLH2908 and OLH2909, which contain 20 nucleotides of *BIM1* genomic sequence preceding an NGG as well as BsmBI-hybridizing sequences sites, were hybridized to create a duplex and cloned into pUB1306 using Golden Gate Assembly ( Figure 1A ; Sawyer et al. 2019; Schlissel and Rine 2019). pXM27 was sequenced (Primordium) to confirm construction.

The repair template for CRISPR was created by first PCR amplifying the GFP variant, *ENVY,* coding sequence from pFA6a-Link-ENVY- *SpHIS5* (Slubowski et al *.* 2014) and adding sequences coding for the linkers GA _5_ and GGGS _2_ on 5’ and 3’ ends respectively, using OLH2904 and OLH2905 (Table 5; linker sequences indicated in lowercase while sequences used for PCR annealing in uppercase). This product was then purified (Monarch, New England Biolabs) and used as the template for PCR, to add 40 base pairs of *BIM1* homology to either side of the insertion point using OLH2906 and OLH2907 (Table 5; *BIM1* homology indicated in lowercase while sequences used for PCR annealing in uppercase); this product was co-transformed into a *MAT* ***a*** wild type yeast strain (LH175) with pXM27 containing the gRNA sequences and plated on selective SD-URA plates.

To screen for appropriate insertion into the *BIM1* locus, colonies were first screened for *ENVY* expression. Fluorescent yeast were confirmed for proper insertion by sequencing: *BIM1* was amplified using OLH2599 and OLH2600 and Sanger sequenced (Quintara) using OLH2601 and OLH2603. These two sequencing primers provide information across the insertion site in both directions.

The strain containing the insertion was first streaked on 5-Fluroorotic Acid (5-FOA) plates to lose pXM27 and then backcrossed to *MATa bim1-myc:HIS5 ^S.p.^* (LH1160) to obtain *MAT* ***a*** and *MATα* strains with the *BIM1* insertion allele (LH1156 and LH1157). These haploids were mated to create a homozygous diploid (LH1158) that was checked for complementation by examining spore formation and germination.

**Table 3. Strains used in this study**

**Table:** 

**Name**	**Genotype**	**Source**
**LH175**	*MAT* ***a*** *ho::LYS2 lys2 ura3 leu2 his3 trp1∆fa*	Huang et al. 2005
**LH176**	*MATα***** *ho::LYS2 lys2 ura3 leu2 his3 trp1∆fa*	Huang et al. 2005
**LH1156**	*MAT* ***a*** *ho::LYS2 lys2 ura3 leu2 his3 trp1∆fa BIM1-S334_N335insENVY*	This study
**LH1157**	*MATα***** *ho::LYS2 lys2 ura3 leu2 his3 trp1∆fa BIM1-S334_N335insENVY*	This study
**LH1158**	*MAT* ***a/*** *α***** *ho::LYS2/ho::LYS2 lys2/lys2 ura3/ura3 leu2/leu2 his3/his3 trp1∆fa/trp1∆fa BIM1-S334_N335insENVY/BIM1-S334_N335insENVY*	This study
**LH1159**	*MAT* ***a*** *ho::LYS2 lys2 ura3 leu2 his3 trp1∆fa bim1-myc:HIS5 ^S.p.^*	Seitz et al. 2023
**LH1160**	*MATα***** *ho::LYS2 lys2 ura3 leu2 his3 trp1∆fa bim1-myc:HIS5 ^S.p.^*	Seitz et al. 2023
**LH1161**	*MAT* ***a/*** *α***** *ho::LYS2/ho::LYS2 lys2/lys2 ura3/ura3 leu2/leu2 his3/his3 trp1∆fa/trp1∆fa bim1-myc:HIS5 ^S.p.^ /bim1-myc:HIS5 ^S.p.^*	Seitz et al. 2023

**Table 4. Plasmids used in this study**

**Table:** 

**Plasmid Descriptor**	**Number/Name**	**Genotype**	**Source**
Cas9 Plasmid	**pUB1306**	CEN/ARS- *URA3* -pPGK1-Cas9-2x SV40 NLS-ptF(GAA)B-gRNA scaffold	Sawyer et al. 2018; Schlissel and Rine 2019
*ENVY* Plasmid	**pFA6a-link-ENVY-SpHIS5**	pFA6a-link-ENVY-SpHIS5	Slubowski et al *.* 2015
Cas9 Plasmid with *BIM1* Guides	**pXM27**	CEN/ARS-URA3-pPGK1-Cas9-2x SV40 NLS-ptF(GAA)B- *BIM1* gRNA	This study

**Table 5. Primers used in this study**

**Table:** 

**Name**	**Sequences (5' to 3')**	**Source**	**Description**
**OLH2908**	gactCAACAACTTGATCATCGACG	This study	Hybridized with OLH2909 to create a duplex containing the gRNA targeting *BIM1* for *ENVY* insertion
**OLH2909**	aaacCGTCGATGATCAAGTTGTTG	This study	Hybridized with OLH2908 to create a duplex containing the gRNA targeting *BIM1* for *ENVY* insertion
**OLH2904**	ggtgctggtgctggtgctggtgctggtgctATGTCTAAAGGCGAGGAATTGTTTACAGGT	This study	Used to amplify *ENVY* with GA _5_ linker
**OLH2905**	agaaccaccaccagaaccaccaccTTTGTACAATTCGTCCATTCCTAATGTTATACCAGC	This study	Used to amplify *ENVY* with GGGS _2_ linker
**OLH2906**	tgtgataatgcagaatgacgaaggtgaggttggcgtgagcGGTGCTGGTGCTGGTGCTGG	This study	Used to amplify *ENVY* with linkers and add *BIM1* homology
**OLH2907**	tctcaacttaaaaagtttcttcgtcgatgatcaagttgttAGAACCACCACCAGAACCAC	This study	Used to amplify *ENVY* with linkers and add *BIM1* homology
**OLH2599**	GTACGCTCGAGTTTACCAT	This study	Used to amplify *BIM1* for sequencing
**OLH2600**	CAAAGAGCAATACCGAACC	This study	Used to amplify *BIM1* for sequencing
**OLH2601**	CCTGGTCCATACCACTCAAG	This study	Used to sequence *BIM1*
**OLH2603**	CGGTGGCTTCTTCATCTCAC	This study	Used to sequence *BIM1*
